# Gyosaponin ameliorates sevoflurane anesthesia-induced cognitive dysfunction and neuronal apoptosis in rats through modulation of the PI3K/AKT/mTOR pathway

**DOI:** 10.1016/j.clinsp.2024.100560

**Published:** 2024-12-20

**Authors:** Lijuan Lin, Chenhui Zhu, Bing Yan, Pinxian Yu, Liu Yang, Wei Huang, Junren Chen

**Affiliations:** a Department of Anesthesia, Central People's Hospital of Zhanjiang, Zhanjiang City, Guangdong Province, PR China; b Department of Urology, Central People's Hospital of Zhanjiang, Zhanjiang City, Guangdong Province, PR China

**Keywords:** Gyosaponin, Sevoflurane, Cognitive Dysfunction, Neuronal Cell Apoptosis, PI3K/AKT/mTOR Pathway

## Abstract

**Background:**

Sevoflurane (Sev) is an inhalational anesthetic for surgical procedures where it can trigger cognitive dysfunction and neuronal apoptosis. Gyosaponin (GpS) was studied for its effects on brain morphology and cognitive behaviors in Sev-anesthetized rats.

**Methods:**

Male Sprague-Dawley rats were induced by 3 % Sev anesthesia, and 25 mg/kg and 100 mg/kg GpS were injected into the rats by tail vein. The in vitro model of Sev anesthesia was constructed by treating primary rat hippocampal neurons with 4.1 % Sev in the presence of GpS (5, 10, and 20 μM). The neuroprotective effects of GpS against Sev-induced cognitive deficits in rats were evaluated using the open field and Morris water maze tests. The apoptosis of hippocampal neurons was observed using HE staining and TUNEL assay. Apoptosis-related proteins and proteins related to the PI3K/Akt/mTOR pathway were determined via Western blot. Also, pro-inflammatory factors were measured via ELISA.

**Results:**

GpS diminished the Sev-triggered apoptosis in neurons and Cleaved caspase-3, BAX, TNF-α, IL-6, lessened oxidative stress damage, and stimulated the PI3K/Akt/mTOR pathway. GpS therapy markedly enhanced learning and memory abilities in rats suffering from Sev-related cognitive impairments.

**Conclusion:**

GpS ameliorates Sev-induced neurotoxicity and cognitive dysfunction by modulating the PI3K/Akt/mTOR pathway and alleviating neuronal apoptosis and oxidative stress.

## Highlights

• GpS ameliorates Sev-induced cognitive deficits in rats.
• GpS ameliorates Sev-induced neuronal cell damage in rats.
• GpS promotes activation of the PI3K/AKT/mTOR pathway.
• PI3K/AKT/mTOR pathway inhibitor reduces the amelioration of cognitive deficits in Sev-anesthetized rats by high-dose GpS.
• PI3K/AKT/mTOR pathway inhibitor impairs the ameliorative effect of high-dose GpS on neuronal cell damage in Sev-anesthetized rats.

## Introduction

Sevoflurane (Sev) is an inhalation anesthetic for surgical procedures and is characterized by a rapid onset of anesthesia and a short recovery time.^1^^,^^2^ Sev can produce toxic effects on neurons,^3^ which may be mediated through neuroinflammation, neurotransmitter imbalance, or decreased concentrations of brain-derived neurotrophic factor.^4^, ^5^, ^6^, ^7^ A further problem is that anesthesia affects the production of fast synapses due to its neurotoxicity. Meanwhile, Sev has been demonstrated to cause postoperative cognitive dysfunction.^8^ Persistent exposure to Sev impairs memory and learning in aged male rats, along with cognitively relevant biochemical changes in the hippocampal region.^9^ Neurodegenerative diseases can impair cognitive function, in which inflammation plays an important role.^10^^,^^11^ Sev is known to increase IL-6 and TNF-α, causing neuroinflammation^12^ in postoperative cognitive dysfunction.^13^^,^^14^ Learning and memory are aided by the hippocampus of the brain.^15^ Several studies have shown that Sev treatment reduces hippocampal neuronal viability but contributes to apoptosis and oxidative stress^3^^,^^16^ and increases ROS levels.

Gynostemma pentaphyllum Makino is a common folk medicine in China, and dammarane saponins are isolated from GP.^17^ Primarily, Gyosaponin (GpS) serves as a remedy for hyperlipidemia, heart-related illnesses, and persistent inflammation,^18^ offering diverse pharmacological benefits like neuroprotection, heart protection, anti-aging, and anti-cancer properties.^19^, ^20^, ^21^, ^22^ GpS exerts antioxidant capacity in glutamate-treated rat cortical cells, as well as in the hippocampal region and cortex of a rat model of chronic cerebral hypoperfusion.^23^^,^^24^ GpS exerts neuroprotective effects on neurons in primary substantia nigra cultures.^25^^,^^26^ Also, GpS can improve oxidative neuronal damage and inflammation after chronic cerebral underperfusion.^24^

Phosphatidylinositol 3-Kinase (PI3K)/protein Kinase B (AKT) signaling is one of the major pro-survival pathways involved in the regulation of a variety of biological response processes.^27^ Inflammatory cytokine release is mediated by the PI3K/Akt/mTOR cascade reaction.^28^^,^^29^ Activation of mTOR prevents inflammation by inhibiting NF-κB activation.^30^ Furthermore, research indicates a heightened release of pro-inflammatory cytokines post-Sev anesthesia, coinciding with the deactivation of the PI3K/Akt/mTOR pathway, hinting at a potential link between this pathway and neuroinflammation induced by Sev anesthesia.^31^ Phosphorylated expression of the PI3K/AKT/mTOR pathway is mainly involved in synaptogenesis, corticogenesis, and related neuronal brain processes, especially in the hippocampus.^32^ Li et al. found that GpS binds well to PI3K, AKT, and mTOR by network pharmacological analysis to induce apoptosis in bladder cancer cells.^33^

Given Sev-induced inflammation and apoptosis and the anti-inflammatory properties of GpS, the authors aimed to determine whether GpS protects hippocampal neurons from Sev anesthesia-induced inflammation and apoptosis and to determine whether these protective effects are closely linked to the PI3K/Akt/mTOR pathway.

## Materials and methods

### Construction of rat model

A group of thirty Sprague-Dawley (SD) rats (male, aged 8 months, weighing 280‒320 g), acquired from Beijing Vital River Laboratory Animal Technology Co., Ltd., received food and water in a pathogen-free environment and were kept in a temperature range of 22‒24 °C with a 12 hour light/dark rhythm. All animal surgeries were approved by the Central People's Hospital of Zhanjiang. The rats were divided into the following six groups: control, Sev, Sev + Low GpS, Sev + High GpS, Sev + Low GpS + LY294002, and Sev + High GpS + LY294002.

Rats were given GpS (Sigma-Aldrich; purity ≥98 %) via tail vein injection 1h before Sev exposure at a low dose of 25 mg/kg and a high dose of 100 mg/kg.^34^ The same dose of saline was injected into the control group.

One hour after the GpS injection, every rat was situated in a chamber designed for inducing anesthesia. The levels of oxygen and the dosage of anesthesia were under constant observation. Treatment of the control group involved 60 % oxygen for 2h, while the Sev group received a treatment of 3 % Sev (Jiangsu Hengrui Pharmaceuticals Co., Ltd., China) for 2 h by inhalation. Anesthesia was initiated at the time when the Sev concentration reached the maximum value. The gas flow rate in the anesthesia room was maintained at 4 L/min.^31^ After 48 h, the rats gradually recovered and were tested for\ behavioral experiments.

To investigate the effect of inhibition of the PI3K/AKT/mTOR pathway on Sev-induced cognitive dysfunction in rats, 5 mL of 5 μg/μL of the PI3K-specific inhibitor LY294002 (Sigma-Aldrich, USA) or control vector (5 μL 10 % DMSO) was injected into left lateral ventricle at 2.5 μL/min using a 5 μL Hamilton syringe (stereotactic coordinates: 2.0 mm rostral and 1.5 mm lateral λ and 2.0 mm deep to the cranial surface).^35^

At the end of the behavioral experiment, the rats were anesthetized and executed with sodium pentobarbital injections intraperitoneally (Sigma-Aldrich). Brain tissues were placed on aluminum foil and hippocampal tissues were separated and stored at -80 °C.

### Morris Water Maze (MWM) Test

In an opaque pool of water 60 cm high and 150 cm in diameter, there are 4 quadrants. The pool was located in an isolated room covered by a black curtain. At the center of the left-right quadrant, a clear platform measuring 10 cm across was installed, with the water rising 1 cm above the platform's height. The water's temperature was kept steady at 20 °C, and it was coated with bleach. Suspended over the pool, a video camera equipped with active tracking software was used to record the path of the rat's movement. Every rat was submerged in water from four distinct quadrants and tallied for a duration of 60s.

The location of the platform (where rats remained for over 2s) was documented. In cases where the platform remained undiscovered after 60s, the rats were directed to locate it and remained on it for 15s. Each rat underwent testing four times daily (one in each quadrant) over a span of 5 days. The average escape latency was gauged as a measure of learning capacity. The examination commenced on the 6th day. Following the training session, the platform was taken out of the pool, and every rat was relocated to the opposite quadrant for 60s. To monitor the duration of the rats' stay in the designated quadrant, their frequency of crossing the platform in under 60s, and their speed of swimming, a video tracking system was employed.

### Open-field test

The rats resided in a square-shaped plywood box, coated with black lacquer enamel, measuring 72 cm long, 30 cm tall, and 72 cm wide. To mimic the conditions of day and night, synthetic lighting and darkness were employed. A photographic device was affixed to monitor the rats' mean traversal distance, their residence time, travel trajectories, and the duration they remained in the central zone for 5 min. The EthoVision XT trajectory tracking system software (version 17.0; Noldus, Netherlands) was utilized to monitor the travel distance and residence time in the central region.

### HE staining

Tissues from the hippocampus were preserved in 4 % paraformaldehyde for a day, dehydrated, encased in paraffin, sliced coronally at a thickness of 3 μM, and dyed with HE staining (Beyotime). Differentiation of the sections occurred using 1 % hydrochloric acid in alcohol for 10 min, followed by a 10-second rinse with 2 % sodium bicarbonate (Beyotime), and a subsequent 3-minute staining with eosin. Following the sections' dehydration using a series of alcohol concentrations, they underwent a rinse with xylene (214,736; Sigma-Aldrich) and were subsequently sealed using neutral resin. The pathological alterations in brain tissue were observed through a microscope (Olympus BX 53 microscope, Tokyo, Japan).

### TUNEL assay

Hippocampal tissues were sectioned to 5 μM using the DeadEnd™ Fluorescent TUNEL System (Promega, WI, USA). Cell nuclei were restained using Hoechst stain and analyzed using NIS-Elements software (Nikon, Tokyo, Japan).

### Immunofluorescence

Tissue sections were rinsed with PBS, fixed with 4 % paraformaldehyde, and permeabilized with 0.5 % Triton X-100. After 5 % BSA for 30 min, sections were incubated with Cleaved Caspase-3 (Cell Signaling Technology; #9661) overnight at 4 °C and Alexa Fluor 488 and Alexa Fluor 555-labeled IgG for 30 min at 37 °C. Nuclei were stained with DAPI and the results were observed by light microscopy.

### Cell culture and treatment

Primary rat hippocampal neurons (Procell Life Science & Technology, Wuhan, China) were cultured routinely (5 % CO_2_, 37 °C) in Dulbecco's modified Eagle's medium/F12 (51445C; Sigma-Aldrich, USA) containing 2 % B-27 and 10 % FBS (12103C; Sigma-Aldrich).

Mimicking anesthetized cell conditions, hippocampal neurons underwent a 6-hour treatment with 4.1 % Sev (Y0001046; Sigma-Aldrich) and GpS at 5, 10, or 20 μM (PHL83847; Sigma), followed by a 24-hour culture. Subsequently, GpS was mixed into DMSO (D2650; Sigma-Aldrich) and diluted using PBS. Hippocampal neurons in both the control and Sev groups received an identical amount of the vector.^36^

Evaluating the impact of PI3K/AKT/mTOR on Sev-anesthetized hippocampal neurons involved pre-treating Sev-induced neurons for 24h with 10 μM of the PI3K-specific inhibitor LY294002.

### MTT assay

After Sev treatment, hippocampal neurons were cultured in 96-well plates with or without GpS (5, 10, or 20 μM) for 24h These cells were exposed to 5 mg/mL MTT solution (20 μL/well; 11,465,007,001; Sigma-Aldrich) for 4h and added with 150 μL of dimethylsulfoxide (Sigma Aldrich) for 10 min. The absorbance values at 490 nm were detected using a microplate reader (Thermo Fisher Scientific, USA).

### Flow cytometry

The apoptosis rate was assessed by Annexin V-FITC/PI Apoptosis Detection kit (Invitrogen). Hippocampal neurons (1 × 10^5^) were washed twice with pre-cooled PBS, resuspended in 500 μL of 1 × Binding Buffer, and 5 μL each of Annexin V-FITC and propidium iodide were added, respectively, and the percentage of apoptotic cells was analyzed by using a FACScan® flow cytometer (BD Biosciences, USA) after staining for 30 min.

### ROS detection

ROS was detected using the ROS detection kit (Nanjing Jinacheng, China, E004–1-1). Hippocampal tissues were made into sections of approximately 1 mm^3^ and enzymatically treated at 37 °C for 30 min. Subsequently, hippocampal neurons were centrifuged at 500 g for 10 min and incubated with 500 µL of 10 µM DCFH-DA in 1 × PBS solution at 37 °C for 30 min in the dark. Post-incubation, the fluorescence of 2,7-dichlorofluorescein was gauged with a NovoCyte 3000 flow cytometer (ACEA Biosciences, USA). The quantity of ROS was denoted by the average value of fluorescence intensity. The ACEA NovoExpress software was utilized for analyzing the samples. Images were taken under a fluorescence microscope (Lecia).

### Oxidative stress assay

MDA content, SOD activity, and GSH content were measured by commercial kits (Abcam, USA).

### Western blot

Hippocampal neurons and hippocampal tissue samples were washed twice with pre-cooled PBS and lysed on ice for 20 min using RIPA lysis buffer (Vazyme, FD008). Protein concentration was assayed using the Pierce ALI Protein Assay Kit (Rockford). Proteins were separated using 10 % SDS-PAGE, transferred to PVDF membranes (Millipore), and blocked with 5 % skimmed milk for 2 h Specimens were incubated with primary antibodies against PI3K (Abcam; ab302958), p-PI3K (Thermo Fisher; PA5–104,853), Akt (Abcam; ab38449), p-Akt (CST; 9271S), mTOR (Abcam; ab134903), p-mTOR (CST; 2971S), Cleaved Caspase-3 (CST; #9661), Bax (Abcam; ab32503), Bcl-2 (Abcam; ab182858), and GAPDH (Abcam; ab37168) overnight at 4 °C. The secondary antibody (Abcam; ab205719) was detected for 1h at 37 °C. Finally, the results were visualized with the Enhanced Chemiluminescence Detection Kit (Vazyme, E411–04) and checked on the FluorChem™M system.

### ELISA assay

TNF-α and IL-6 concentrations in hippocampal tissue and hippocampal neurons were determined using ELISA Kits (R&D Systems, USA).

### Data analysis

The experimental data were statistically analyzed using SPSS20. Data were expressed as mean ± SD. The *t*-test was employed for comparison between two groups, and one-way variance for that between multiple groups (* p < 0.05 indicated that the difference was statistically significant).

## Results

### GpS ameliorates Sev-induced cognitive deficits in rats

**Fig. 1:**
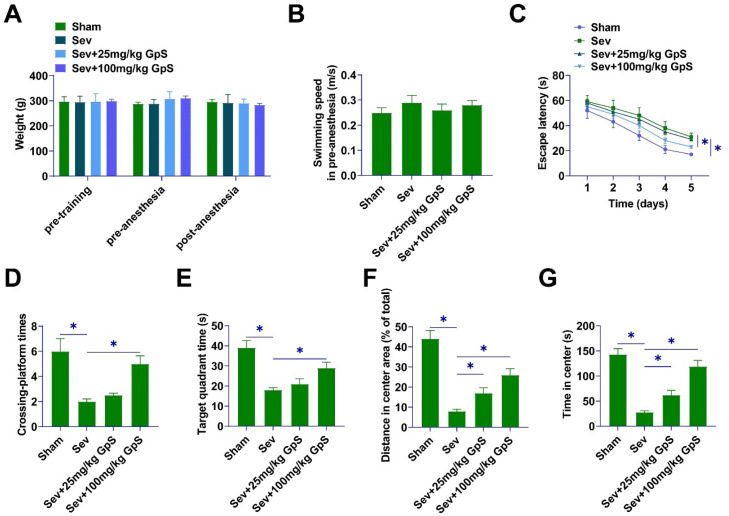
**GpS ameliorates Sev-induced cognitive deficits in rats.** (A) Body weight of rats in each group; (B) Swimming speed of rats; (C) Escape latency of rats; (D) Number of times traversing the platform in 60s; (E) Time spent in the target quadrant; (F) Distance traveled by rats; (G) Time spent by rats in the central area. Data are expressed as mean ± SD (n = 5). * p < 0.05.

MWM test was conducted on rats to detect the effects of GpS on the cognitive abilities of rats affected by Sev anesthesia. Neither pre-training nor pre-anesthesia nor post-anesthesia rats' body weights differed significantly (Fig. 1A). Twenty-four hours after Sev anesthesia, the rats in the Sham group showed clear swimming paths, suggesting that their recognition ability was not affected. In addition, there was no statistically significant difference in the swimming speed of the rats in each group (Fig. 1B). Importantly, the authors observed an increase in the escape latency of rats after Sev anesthesia, but this change was attenuated by high-dose GpS treatment (Fig. 1C). As a result, the number of times rats in the Sev group traversed the platform and the residence time in the target quadrant were lower than those in the Sham group, whereas the high-dose GpS treatment attenuated this effect, and the low-dose GpS had almost no ameliorating effect (Fig. 1D‒E). Open field experiment indicated that Sev-anesthetized rats traveled a distance shorter than that of Sham-group rats, while rats treated with high doses of GpS exhibited similar behaviors to those of Sham-group rats (Fig. 1F). Sev-anesthetized rats stayed at the center for a significantly longer period of time, whereas high-dose GpS significantly shortened it (Fig. 1G).

### GpS ameliorates Sev-induced neuronal cell damage in rats

**Fig. 2:**
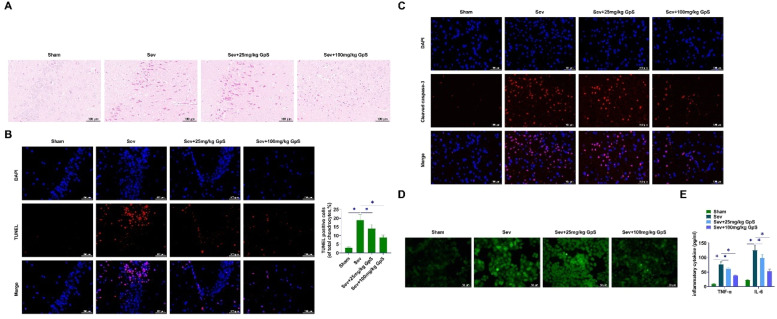
**GpS ameliorates Sev-induced neuronal damage in rats.** (A) HE staining to assess neuronal damage in rats; (B) TUNEL to assess apoptosis in rat neurons; (C) Immunofluorescence to measure Cleaved caspase-3 expression in rat hippocampal tissues; (D) Measurement of ROS production in hippocampal neurons using fluorescence microscope; (E) ELISA to determine TNF-α and IL-6 in rat hippocampal tissues. Data are expressed as mean ± SD (n = 5). *p < 0.05.

HE staining demonstrated that hippocampal neurons in Sham-operated rats were neatly arranged with intact cell structures and regular and large nuclei. Sev-anesthetized rats showed chromatin condensation and nucleation, indicating neuronal damage and increased apoptosis. Rats treated with high doses of GpS showed neatly arranged cells with intact cellular structures, which had an ameliorating effect on the damage (Fig. 2A). TUNEL assay showed a significant increase in the number of positive cells in Sev-anesthetized rats, and a certain inhibitory effect on neuronal apoptosis by low-dose GpS, while the high-dose GpS treatment significantly decreased the hippocampal positive cell number (Fig. 2B). Cleaved caspase-3 in Sev-anesthetized rats was decreased by high dose GpS treatment as determined by immunofluorescence (Fig. 2C). Fluorescence microscope indicated that ROS production was increased by Sev induction, whereas high-dose GpS attenuated this effect and mitigated oxidative stress injury (Fig. 2D). Finally, ELISA assay showed that high-dose GpS significantly inhibited the promotion of TNF-α and IL-6 production by Sev in hippocampal tissues and attenuated the inflammatory response (Fig. 2E).

### GpS promotes activation of the PI3K/AKT/mTOR pathway

**Fig. 3:**
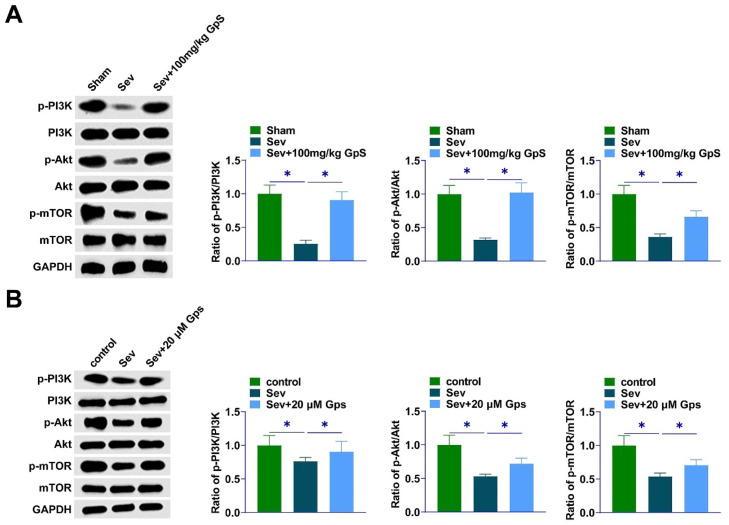
**GpS promotes the activation of PI3K/AKT/mTOR pathway.** (A) Western Blot assay to determine proteins related to PI3K/Akt/mTOR pathway in rat hippocampal tissues; (B) Western Blot assay to determine proteins related to PI3K/Akt/mTOR signaling pathway in primary rat hippocampal neurons. Data are expressed as mean ± SD (n = 3). *p < 0.01.

Western Blot detected lower p-PI3K, p-Akt, and p-mTOR in the hippocampal region of Sev-anesthetized rats. However, GpS could reverse this effect (Fig. 3A). In addition, consistent results were also exhibited in primary rat hippocampal neurons anesthetized by Sev (Fig. 3B).

### PI3K/AKT/mTOR pathway inhibitor reduces the amelioration of cognitive deficits in Sev-anesthetized rats by high-dose GpS

**Fig. 4:**
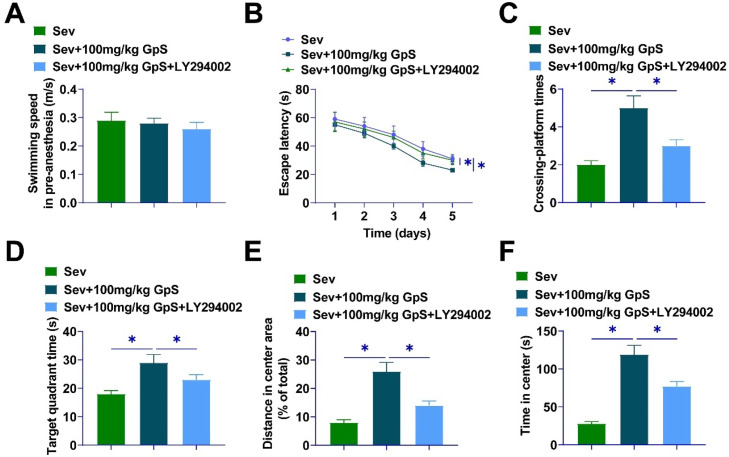
**PI3K/AKT/mTOR pathway inhibitor reduces the amelioration of cognitive deficits in Sev-anesthetized rats by high-dose GpS.** (A) Swimming speed of rats; (B) Escape latency of rats; (C) Number of times crossing the platform in 60s; (D) Time spent in the target quadrant; (E) Distance traveled by rats; (F) Time spent in the center by rats. Data are expressed as mean ± SD (n = 5). *p < 0.05.

The PI3K-specific pathway inhibitor LY294002 was employed to treat Sev-anesthetized rats. After MWM experiments, the swimming speed did not differ significantly between groups (Fig. 4A), the escape latency of the rats under the effect of GpS was shortened, and the number of times crossing the platform and the residence time in the target quadrant was prolonged, which were mitigated by LY294002 (Fig. 4B‒D). GpS significantly prolonged the distance traveled and the central residence time of rats, whereas LY294002 treatment significantly impaired the effects of GpS (Fig. 4E‒F).

### PI3K/AKT/mTOR pathway inhibitor impairs the ameliorative effect of high-dose GpS on neuronal cell damage in Sev-anesthetized rats

**Fig. 5:**
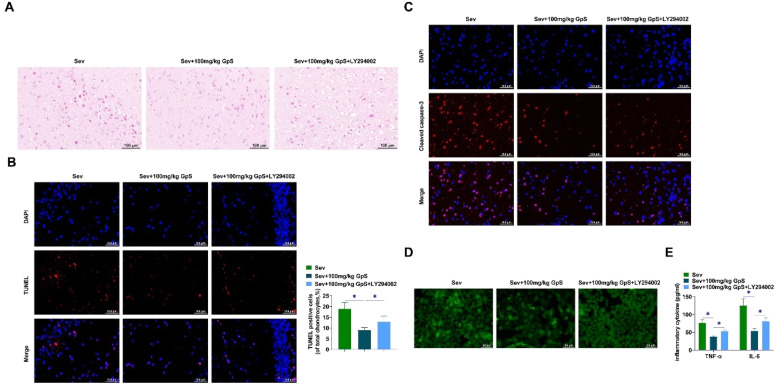
**PI3K/AKT/mTOR pathway inhibitor impairs the ameliorative effect of high-dose GpS on neuronal cell damage in Sev-anesthetized rats.** (A) HE staining to assess neuronal damage in rats; (B) TUNEL to assess apoptosis in rat neurons; (C) Immunofluorescence to measure Cleaved caspase-3 expression in rat hippocampal tissues; (D) Measurement of ROS production in hippocampal neurons using fluorescence microscope; (E) ELISA to determine TNF-α and IL-6 in rat hippocampal tissues. Data are expressed as mean ± SD (n = 5). *p < 0.05.

HE staining revealed that high-dose GpS-treated rats showed neatly arranged cells with intact cellular structures, whereas LY294002 treatment attenuated the effects of GpS, and the neurons were significantly damaged (Fig. 5A). TUNEL assay results showed that LY294002 impaired the action of high-dose GpS treatment on the number of hippocampus-positive cells in rats (Fig. 5B). High-dose GpS treatment decreased Cleaved caspase-3 in the Sev group as shown by immunofluorescence assay, and LY294002 mitigated the effect (Fig. 5C). High-dose GpS attenuated ROS production, treatment with LY294002 significantly increased ROS content (Fig. 5D). LY294002 weakened the impact of high-dose GpS on TNF-α and IL-6 production (Fig. 5E).

### GpS ameliorates damage in Sev-anesthetized primary hippocampal neurons

**Fig. 6:**
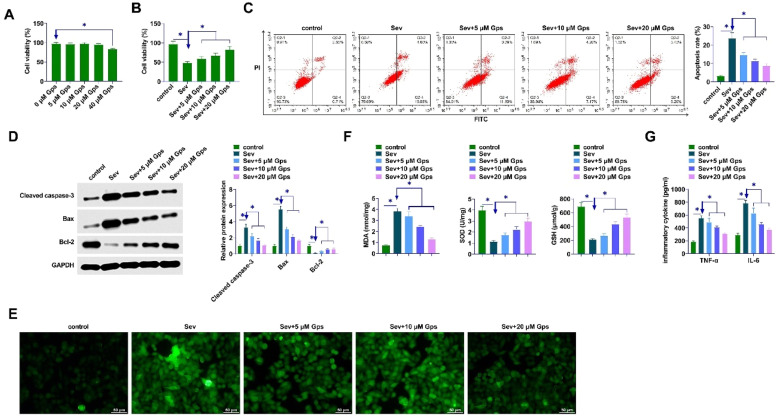
**GpS ameliorates the damage of Sev-anesthetized primary hippocampal neurons.** (A) Cytotoxicity of GpS; (B) Viability of hippocampal neurons detected by CCK-8; (C) Apoptosis of hippocampal neurons detected by flow cytometry; (D) Expression of Cleaved caspase-3, Bax, and Bcl-2 determined by Western Blot; (E) Measurement of intracellular ROS levels in primary hippocampal neurons; (F) MDA, SOD, and GSH levels were measured by commercial kits; (G) ELISA determination of TNF-α and IL-6 in hippocampal neurons. Data are expressed as mean ± SD (n = 3). *p < 0.01.

The cytotoxicity of GpS was confirmed by exposing hippocampal neurons to different concentrations (0, 5, 10, 20, or 40 μM). The viability of hippocampal neurons was reduced after 40 μM GpS treatment (Fig. 6A). CCK-8 assay demonstrated that Sev treatment decreased the viability of hippocampal neurons, whereas GpS treatment enhanced hippocampal neuronal viability dose-dependently (Fig. 6B). Flow cytometry demonstrated that Sev treatment increased hippocampal neuronal apoptosis, while GpS inhibited hippocampal neuronal apoptosis dose-dependently (Fig. 6C). GpS inhibited Cleaved caspase-3 and Bax and promoted Bcl-2 dose-dependently as shown by Western Blot assay (Fig. 6D). Moreover, GpS attenuated the oxidative stress damage in hippocampal neurons by attenuating the ROS level and the content of MDA, SOD, and GSH (Fig. 6E‒F). Also, ELISA assayed that Sev anesthesia promoted TNF-α and IL-6 production in hippocampal neurons, whereas GpS attenuated TNF-α and IL-6 contents (Fig. 6G).

### PI3K/AKT/mTOR pathway inhibitor weakens the ameliorative effect of high-dose GpS on cell damage in Sev-anesthetized primary hippocampal neurons

**Fig. 7:**
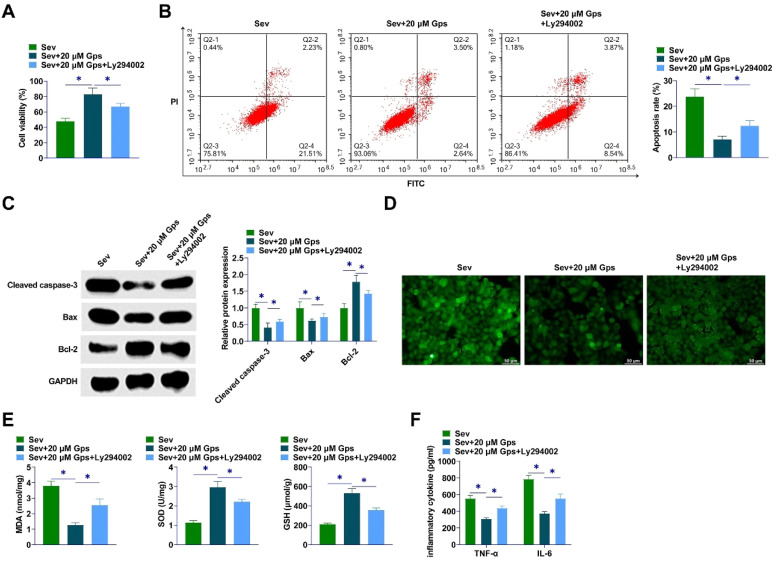
**PI3K/AKT/mTOR pathway inhibitor weakens the ameliorative effect of high-dose GpS on cell damage in Sev-anesthetized primary hippocampal neurons.** (A) Viability of hippocampal neurons detected by CCK-8; (B) Apoptosis of hippocampal neurons detected by flow cytometry; (C) Expression of Cleaved caspase-3, Bax, and Bcl-2 determined by Western Blot; (D) Measurement of intracellular ROS levels in primary hippocampal neurons; (E) Levels of MDA, SOD, and GSH determined by commercial kits assay; (F) ELISA determination of TNF-α and IL-6 levels in hippocampal neurons. Data are expressed as mean ± SD (n = 3). * p < 0.01.

The PI3K-specific signaling pathway inhibitor LY294002 was used to treat Sev-anesthetized hippocampal neurons. LY294002 reduced the promotional effect of GpS on the viability of hippocampal neurons (Fig. 7A). Flow cytometry assayed that GpS inhibited apoptosis of hippocampal neurons, while LY294002 prevented this effect (Fig. 7B). GpS inhibited Cleaved caspase-3 and Bax and promoted Bcl-2, while LY294002 blocked this effect (Fig. 7C). Then, LY294002 impaired the inhibitory effect of GpS on ROS levels and MDA, SOD, and GSH contents and aggravated oxidative stress injury in hippocampal neurons (Fig. 7D‒E). Moreover, GpS attenuated TNF-α and IL-6 content, while LY294002 weakened this effect (Fig. 7F).

## Discussion

Clinical practitioners use Sev because of its early awakening time. In addition, it has the advantage of aroma and low irritation of the airways.^37^ When aged male rats are repeatedly exposed to Sev, they experience a decline in learning and memory as well as biochemical changes to the hippocampal region.^9^ It has been documented that human and animal subjects exposed to Sev-based anesthetics exhibit long-term cognitive deficits as well as neuropathological changes in the brain.^38^ According to this study, GpS modulated downstream PI3K/AKT/mTOR pathway to reduce hippocampal inflammation and improve memory, learning, and cognitive function in rats.

It is well known that Sev induces cognitive dysfunction and neuroinflammation postoperatively.^39^ Repeated Sev exposure can lead to cognitive deficits in young rats.^40^^,^^41^ Therefore, the authors constructed a rat model using 3 % Sev inhalation for 2h Exposure at this dose does not cause hypoxia or respiratory depression.^42^^,^^43^ Sev induces neuroinflammation by increasing IL-6 and TNF-α, which can lead to memory and learning deficits.^44^ The results presented that Sev induced apoptosis in the hippocampal neurons of rats. The adult rat brain is highly susceptible to different doses of anesthetic-induced neurotoxicity, leading to cognitive dysfunction.^45^ MWM test has been applied to assess spatial and memory ability.^46^ Shorter escape latencies and an increased number of original platform position crossings indicate better learning and memory abilities. The present results showed that cognitive function was significantly reduced in Sev-anesthetized rats, and HE staining indicated severe damage to hippocampal neurons, consistent with a previous study.^47^ However, a high dose of GpS significantly improved the cognitive deficits caused by Sev anesthesia and enhanced learning and memory abilities.

Neuroinflammation and oxidative stress are key factors in post-surgical cognitive impairment. On the one hand, neuroinflammation is a major source of ROS, free radicals, and reactive nitrogen in the activated central nervous system.^48^ In turn, the excess ROS produced destroys biomolecules, alters cellular function and consequently promotes inflammation.^49^ Sev exposure impairs spatial memory in aged rats by increasing inflammation.^50^ Also, inhalation of Sev enhances the release of cytokines in the hippocampus of adult rats.^51^ Increasing evidence suggests that excessive ROS-induced oxidative stress is considered to be a causative factor for Sev-induced neurotoxicity^43^^,^^52^ and a contributing factor to Sev-induced neuronal death.^53^ In the present study, GpS effectively inhibited the occurrence of oxidative stress injury and inflammatory response in hippocampal neurons of Sev-anesthetized rats.

Many cellular processes, including growth and survival, are governed by the PI3K/Akt/mTOR pathway.^54^^,^^55^ The PI3K/Akt pathway stands as a key pathway for survival, and the stimulation of Akt, an anti-apoptotic agent, by PI3K aids in enhancing cell longevity.^56^ Initiating PI3K/Akt signaling pathways has been shown to lower the likelihood of brain injury in models of ischemic stroke.^57^ mTOR is a major effector protein of Akt.^58^ Wang et al. found that Sev anesthesia leads to an increase in the release of cytokines that promote inflammation, and this release is accompanied by the inactivation of the PI3K/Akt/mTOR pathway. LY294002, an inhibitor of the PI3K/Akt pathway, may inhibit this effect.^31^ In this study, In rats, Sev exposure inactivated the PI3K/Akt/mTOR pathway, but GpS reversed this process. In combination with LY294002, GpS was partially reversed in its therapeutic effect.

In summary, GpS significantly reduced neurological deficits in Sev-anesthetized rats. The neuroprotective effect of this may be related to the GpS treatment-induced expression of PI3K/Akt/mTOR and the significant reduction in Sev-induced cell apoptosis and neuroinflammation. In addition to providing new clinical drug treatment strategies and rationales, this research highlights the efficacy of GpS as a treatment for cognitive dysfunction.

## Availability of data and materials

The datasets used and/or analyzed during the present study are available from the corresponding author upon reasonable request.

## Ethics approval

The present study was approved by the Animal experiments were approved by Central People's Hospital of Zhanjiang Animal Experimental Ethics Committee (IACUC-20211015–83). All procedures complied with the National Institutes of Health Guide for the Use of Laboratory Animals.

## Authors’ contributions

Lijuan Lin Provided Conceptualization. Lijuan Lin, Chenhui Zhu and Bing Yan performed the research. Pinxian Yu, Liu Yang and Junren Chen provided help and advice on the experiments. Lijuan Lin, Wei Huang and Junren Chen analyzed the data. Lijuan Lin wrote the manuscript. Lijuan Lin and Junren Chen reviewed and edited the manuscript. All authors contributed to editorial changes in the manuscript. All authors read and approved the final manuscript.

## Funding

Not applicable.

## Declaration of competing interest

The authors declare no conflicts of interest.
